# PEG Minocycline-Liposomes Ameliorate CNS Autoimmune Disease

**DOI:** 10.1371/journal.pone.0004151

**Published:** 2009-01-07

**Authors:** Wei Hu, Josbert Metselaar, Li-Hong Ben, Petra D. Cravens, Mahendra P. Singh, Elliot M. Frohman, Todd N. Eagar, Michael K. Racke, Bernd C. Kieseier, Olaf Stüve

**Affiliations:** 1 Department of Neurology, University of Texas Southwestern Medical Center at Dallas, Dallas, Texas, United States of America; 2 Department of Pharmaceutics, Utrecht Institute for Pharmaceutical Sciences, Utrecht University, Utrecht, The Netherlands; 3 Department of Ophthalmology, University of Texas Southwestern Medical Center at Dallas, Dallas, Texas, United States of America; 4 Center for Immunology, University of Texas Southwestern Medical Center at Dallas, Dallas, Texas, United States of America; 5 Department of Neurology, Ohio State University, Columbus, Ohio, United States of America; 6 Department of Neurology, Heinrich Heine University, Düsseldorf, Germany; 7 Neurology Section, VA North Texas Health Care System, Medical Service, Dallas, Texas, United States of America; National Institutes of Health, United States of America

## Abstract

**Background:**

Minocycline is an oral tetracycline derivative with good bioavailability in the central nervous system (CNS). Minocycline, a potent inhibitor of matrix metalloproteinase (MMP)-9, attenuates disease activity in experimental autoimmune encephalomyelitis (EAE), an animal model of multiple sclerosis (MS). Potential adverse effects associated with long-term daily minocycline therapy in human patients are concerning. Here, we investigated whether less frequent treatment with long-circulating polyethylene glycol (PEG) minocycline liposomes are effective in treating EAE.

**Findings:**

Performing *in vitro* time kinetic studies of PEG minocycline-liposomes in human peripheral blood mononuclear cells (PBMCs), we determined that PEG minocycline-liposome preparations stabilized with CaCl_2_ are effective in diminishing MMP-9 activity. Intravenous injections of PEG minocycline-liposomes every five days were as effective in ameliorating clinical EAE as daily intraperitoneal injections of minocycline. Treatment of animals with PEG minocycline-liposomes significantly reduced the number of CNS-infiltrating leukocytes, and the overall expression of MMP-9 in the CNS. There was also a significant suppression of MMP-9 expression and proteolytic activity in splenocytes of treated animals, but not in CNS-infiltrating leukocytes. Thus, leukocytes gaining access to the brain and spinal cord require the same absolute amount of MMP-9 in all treatment groups, but minocycline decreases the absolute cell number.

**Conclusions:**

Our data indicate that less frequent injections of PEG minocycline-liposomes are an effective alternative pharmacotherapy to daily minocycline injections for the treatment of CNS autoimmune diseases. Also, inhibition of MMP-9 remains a promising treatment target in EAE and patients with MS.

## Introduction

Experimental autoimmune encephalomyelitis (EAE) is an antigen-specific T cell-mediated autoimmune diseases of the central nervous system (CNS), which has long served as an animal model for the human demyelinating disorder multiple sclerosis (MS) [1]. A pathological hallmark of EAE is the presence of perivascular mononuclear cell infiltrates in the brain and spinal cord [1]. In order to egress from the peripheral blood into peripheral tissues, leukocytes have to transverse endothelial barriers, the basement membrane (basal lamina), and parenchymal extracellular matrix (ECM). Matrix metalloproteinases (MMPs) are proteolytic enzymes that mediate leukocyte migration across the blood-brain barrier (BBB), and through ECM [2]–[6].

Minocycline is an oral tetracycline derivative with good bioavailability within the CNS. It was shown previously that minocycline attenuates neuroinflammation, neuropathological changes, and clinical disease severity in EAE [7]–[9]. The biological effects of minocycline in EAE appear to be at least partially mediated through its effect on the expression and biological activity of MMP-9 [8]. Clinical trials of minocycline in patients with MS are ongoing. Systemic administration of minocycline has been associated with numerous, sometimes serious adverse effects.

Liposomes are spherical vesicles that consist of one or more lipid bilayers that surround an aqueous space. Liposomes were developed as drug carriers because of their capability to enclose biological materials, and to deliver them to specific tissues. Long-circulating polyethylene glycol (PEG) liposomes have two interesting pharmacological properties: (1) Following administration, PEG liposomes remain intact in the blood compartment for extended periods of time; (2) PEG liposomes have a high affinity to, and accumulate predominantly within sites of inflammation [10].

The principal goal of this study was to test the treatment efficacy of PEG minocycline-liposomes in EAE, and to study their effect on MMP-9 expression by leukocytes in peripheral lymphoid organs and the CNS.

## Results

### *In vitro* effects of PEG minocycline-liposomes on MMP-9 expression by human PBMCs

**Figure 1:**
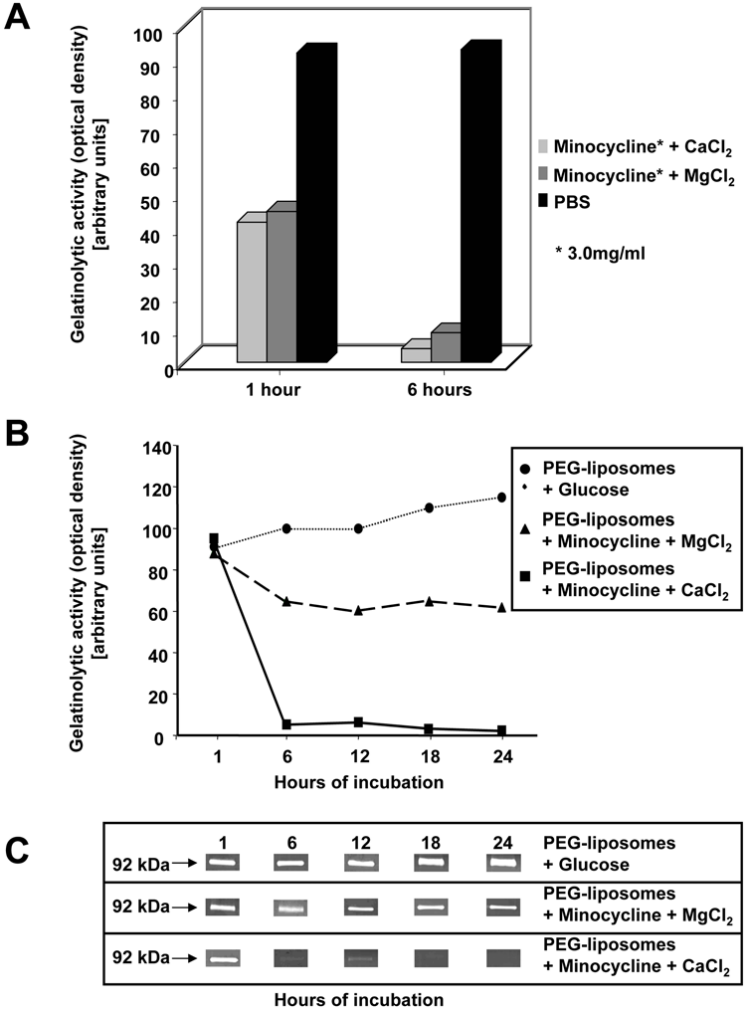
Effects of long-circulating polyethylene glycol (PEG) minocycline-liposomes on the human peripheral blood mononuclear cells (PBMC) in vitro. Quantitative analysis of the zones of gelatinolysis detected the reduced the proteolytic activity of matrix metalloproteinase (MMP)-9 in the human PBMC sample treated with minocycline (3.0 mg/ml) compared with findings in controls (PBS). A more significant decrease of MMP-9 activity was detected in cells treated with minocycline for 6 hours compared to cells that were treated for 1 hour (A). At 1 hour, there was no significant MMP-9 activity difference among samples treated by the different PEG liposome preparation either with glucose, CaCl_2_ or MgCl_2_ (A). Time kinetic studies of PEG minocycline-liposomes *in vitro* revealed that the MMP-9 gelatinolytic activity is significantly reduced at 6 hours after incubation samples with PEG liposome+minocycline (B). Moreover, PEG minocycline-liposome+CaCl_2_ treatment inhibited the MMP-9 activity more strikingly than the PEG minocycline-liposome+MgCl_2_ preparation (B). Gelatinolytic activity was detectable by gelatin-zymography at molecular weights of 92 kDa, indicative of MMP-9, in the supernatants from all human PBMC samples studied. Incubation with PEG minocycline-liposomes with CaCl_2_ resulted in remarkably decreased sizes of the bands at 92 kDa, pointing to decreased activation of MMP-9 in comparison with PEG minocycline-liposomes with MgCl_2_ or glucose, respectively (C).

Proteolytic activity of MMP-9 was assessed in supernatants of human PBMCs after 24 hrs of stimulation with IL-2 and assessed by gelatin-zymography, as previously described [5], [11]. Densitometric analysis of the zones of gelatinolysis revealed a reduced proteolytic activity of MMP-9 in those supernatants derived from PBMCs treated with minocycline (3.0 mg/ml) compared to control (PBS). Specifically, the reduction of MMP-9 proteolytic activity following PEG minocycline-liposomes treatment was more substantial when cells were harvested after 6 hours than after 1 hour, both with PEG minocycline-liposome preparations containing CaCl_2_ or MgCl_2_ (Fig. 1A). When time kinetic studies of PEG minocycline-liposomes were performed *in vitro*, we detected that PEG minocycline-liposomes with CaCl_2_ inhibited the proteolytic activity of MMP-9 more strikingly than PEG minocycline-liposomes with MgCl_2_ or glucose (Fig. 1B&C). Incubation with PEG minocycline-liposomes with CaCl_2_ resulted in a substantial decrease of MMP-9 proteolytic activity compared with PEG minocycline-liposomes with MgCl_2_ or glucose, respectively (Fig. 1C). Consequently, PEG minocycline-liposome preparations containing CaCl_2_ were used in all EAE experiments.

### Effects of PEG minocycline-liposomes on the clinical course of EAE

**Figure 2:**
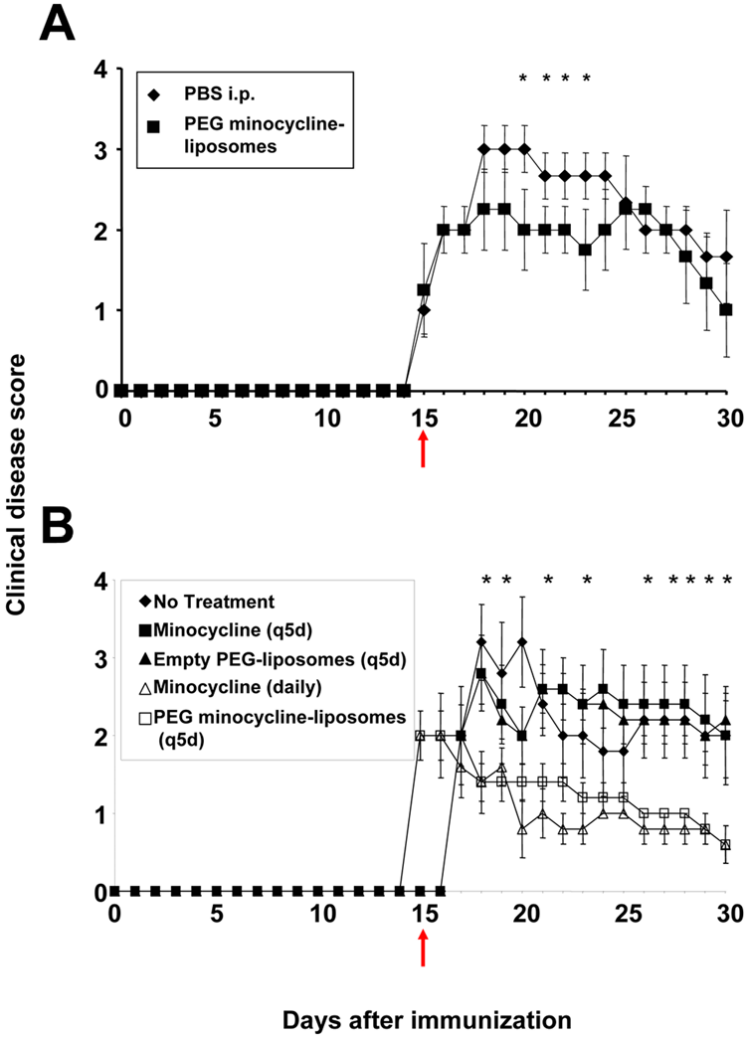
Effects of long-circulating polyethylene glycol (PEG) minocycline-liposomes on the clinical course of experimental autoimmune encephalomyelitis (EAE). A single intravenous (i.v.) injection of PEG minocycline-liposomes given after disease onset at day 15 post-immunization resulted in significant amelioration of clinical disease for eight days compared to a single injection of PBS when given shortly after onset of clinical disease (A). This effect was not sustained (A). In subsequent experiments, PEG-liposomes were administered every five days. Treatment with i.v. PEG minocycline-liposomes initiated after disease onset at day 15 post-immunization and administered every five days was as effective in ameliorating clinical EAE as treatment with daily intraperitoneal (i.p.) injections of minocycline (B). In contrast, i.v. injections of empty PEG-liposome every five days, or minocycline i.p. injections every five days had no detectable effect on the clinical course of EAE (B). The time of treatment initiation is indicated by a red arrow.

To test our hypothesis that less frequent intravenous (i.v.) injections of a liposome formulation have similar therapeutic efficacy as daily injections of regular formulation minocycline, we employed treatment paradigms that did not favor our hypothesis. Other investigators had previously shown that a daily intraperitoneal (i.p.) treatment paradigm in itself may significantly lower clinical disease in mice with EAE, possibly through the release of anti-inflammatory mediators [9]. In initial experiments, a single dose of PEG minocycline-liposomes was administered after EAE disease onset at day 15 post-immunization to determine the duration of efficacy of this preparation. A single i.v. injection of PEG minocycline-liposomes resulted in significant amelioration of clinical disease for eight days compared to a single injection of PBS (Fig. 2A). This beneficial effect was not sustained (Fig. 2A). Consequently, it was decided to administer PEG-liposomes every five days. In clinical practice, the earliest possible time of treatment initiation in patients with MS is after the first clinical attack [12]. Thus, therapy was started at day 15 post immunization, after experimental animals had developed clinical disease. Treatment with i.v. PEG minocycline-liposomes every five days was as effective in ameliorating clinical EAE as treatment with daily i.p. injections of minocycline (Fig. 2B, Table 1). Both treatment paradigms resulted in sustained clinical benefit. In contrast, i.v. injections of empty PEG-liposomes every five days, or minocycline i.p. injections every five days had no detectable effect on the clinical course of EAE (Fig. 2B, Table 1).

**Table 1:** Summary of the EAE disease course^a^.

Treatment	No. of Mice	Incidence^b^ (%)	Mortality^c^ (%)	Average Day of Onset	Average Cumulative Disease Score^d^
No Tx^e^	5	5/5 (100)	0	17	32±6
Mino^f^ (q5d)	5	5/5 (100)	0	17	33±4
Lipo^g^ (q5d)	5	5/5 (100)	1 (20)	17	32±9
Mino^h^ (daily)	5	5/5 (100)	0	15	21±1
Mino-Lipo^i^ (q5d)	5	5/5 (100)	0	15	18±2

a Graded disease score as described in *Materials and Methods*.

b Represents the percentage of mice that developed a clinical score of at least one.

c Represents the percentage of mice that died or were sacrificed for humane purposes.

d The cumulative disease score was calculated by adding the disease score from the day of onset to day 30. The values shown are the mean±SE of all the mice with disease in each group.

e No Tx = No Treatment.

f Mino = Minocycline.

g Lipo = Empty PEG-liposomes.

h Mino = Minocycline.

i Mino-Lipo = PEG minocycline-liposomes.

### Effects of PEG minocycline-liposomes on CNS inflammation

**Figure 3:**
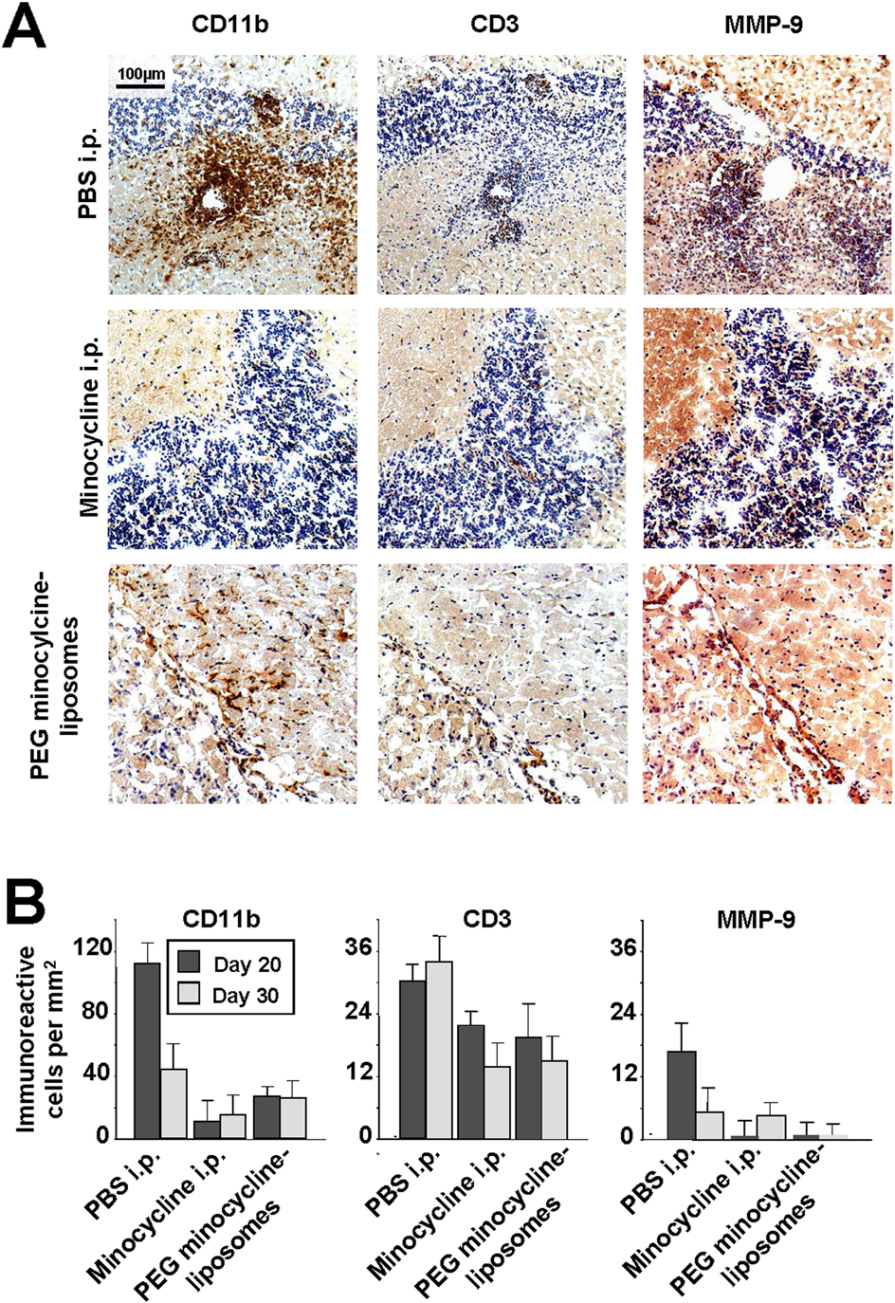
Effects of long-circulating polyethylene glycol (PEG) minocycline-liposomes on inflammation within the central nervous system (CNS). A decreased number of inflammatory foci in the brain of mice with experimental autoimmune encephalomyelitis (EAE) treated with PEG minocycline-liposomes at disease onset was detected (A). Specifically, the number of CD11b^+^ macrophages, CD3^+^ T lymphocytes, and the overall expression of matrix metalloproteinase (MMP)-9 were decreased by PEG minocycline-liposomes treatment (A,B). There was no difference with regard to the numbers of immunoreactive cells between mice that were treated daily with intraperitoneal (i.p.) minocycline, and animals that had received intravenous (i.v.) PEG minocycline-liposomes (B). There was no difference with regard to the number of CD11b^+^ macrophages, CD3^+^ T lymphocytes, and the expression of MMP-9 in the CNS between animals that had received no treatment, daily i.v. PBS injection, and empty PEG-liposomes administered i.p every five days (data not shown).

A decreased number of inflammatory foci in the brain of EAE mice treated with PEG minocycline-liposomes was observed five days after treatment initiation (Fig. 3A). Specifically, the number of CD11b^+^ macrophages, CD3^+^ T lymphocytes, and the overall expression of MMP-9 within CNS tissue was decreased by PEG minocycline-liposomes treatment (Fig. 3A–B). There was no difference with regard to the numbers of immunoreactive cells between mice that were treated daily with i.p. minocycline, and animals treated with i.v. PEG minocycline-liposomes (Fig. 3B).

### Effects of PEG minocycline-liposomes on the expression of MMP-9 in splenocytes and CNS-infiltrating leukocytes

**Figure 4:**
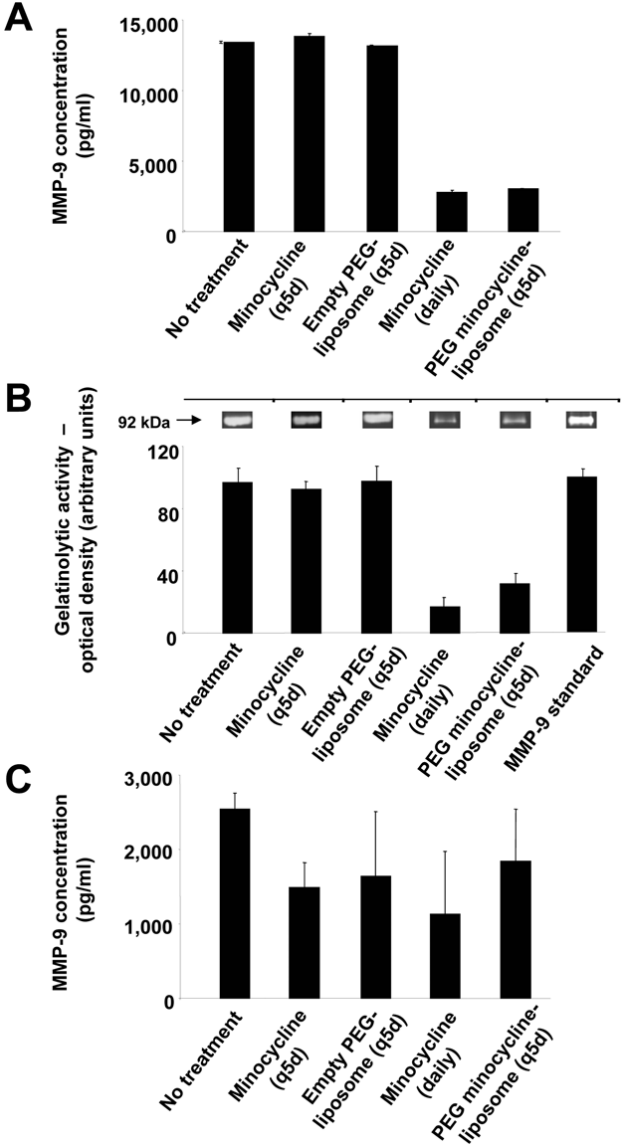
Effects of PEG minocycline-liposomes on the expression of matrix metalloproteinase (MMP)-9 in splenocytes and CNS-infiltrating leukocytes. The expression levels of MMP-9 were significantly down-regulated on day 20 after active induction of experimental autoimmune encephalomyelitis (EAE) in splenocytes of mice following treatment with daily intraperitoneal (i.p.) injections of minocycline, and after intravenous (i.v.) injections of PEG minocycline-liposomes every 5 days compared to experimental animals that received no treatment, that were treated with i.v. injections of empty PEG-liposome every five days, or with minocycline i.p. injections every five days, as shown by ELISA (A). The proteolytic activity of MMP-9 measured by zymography was also significantly diminished in mice treated daily with i.p. injections of minocycline and i.v. injections of PEG minocycline-liposomes every five days (B). MMP-9 protein expression in CNS mononuclear cells was not found to be significantly different between experimental groups (C).

The expression of MMP-9 by splenocytes was significantly reduced in mice treated daily with i.p. injections of minocycline and i.v. injections of PEG minocycline-liposomes every five days compare to splenocytes from animals that received no treatment, i.p. injections of minocycline every five days, and i.v. injections of empty PEG-liposomes every five days, as shown by ELISA (Fig. 4A). As expected, MMP-9 proteolytic activity measured by zymography was also significantly diminished in mice treated daily with i.p. injections of minocycline and i.v. injections of PEG minocycline-liposomes every five days (Fig. 4B). The protein expression of MMP-9 by CNS-infiltrating leukocytes was similar in all treatment groups (Fig. 4C). There was no significant difference with regard to the number of CD11b^+^ macrophages, CD3^+^ T lymphocytes, and the expression of MMP-9 in the CNS between animals that had received no treatment, daily i.p. PBS injection, and empty PEG-liposomes administered i.v. every five days (data not shown).

## Discussion

Minocycline, a semisynthetic tetracycline, is used for the treatment of pneumonia, rheumatoid arthritis (RA), acne, and other infectious diseases. Furthermore, it has been successfully tested in animal models of neurodegeneration, CNS inflammation, and traumatic brain injury [13]. It is currently thought that the clinical benefits achieved by minocycline are the result of caspase-activated apoptosis, and its modulation of peripheral immunocompetent cells and microglia with regard to their release of cytokines, suppression of free radical production, and inhibition of MMPs [13]. More recent studies suggest that minocycline treatment may worsen some diseases of the CNS [13] and the peripheral nerve [14]. Thus, minocycline might have therapeutic potential in neuroinflammatory disorders. However, long-term safety and efficacy need to be determined critically when considering its application in clinical neurology.

Minocycline has an acceptable side-effect profile and tolerability when utilized for short-term antibiosis. Potential adverse effects associated with long-term daily minocycline therapy for patients with MS are concerning. These potential side effects have perhaps most frequently been observed in patients with RA. In a randomized, double-blind study of minocycline in patients with active RA, patients received a maximal oral daily dose of 200 mg for 26 weeks [15]. Six out of 40 patients stopped medication because of adverse events in the minocycline treatment group. Specifically, gastrointestinal adverse effects and dizziness were among the adverse events significantly increased in the minocycline group compared to those patients receiving placebo. Other adverse events that were more frequent in the minocycline treatment group included rash and headaches. Long-term exposure to minocycline has also been associated with a variety of clinical and serological autoimmune aberrations, including serum sickness [16], drug-induced lupus [17], autoimmune hepatitis [18], and vasculitis [19].

Some of the observed adverse events are not entirely surprising. Minocycline is a small (495 kDa), highly lipophilic molecule capable of crossing the blood–brain barrier (BBB). Because of the large volume of distribution of minocycline, the drug accumulates in tissues other than the CNS, including the eye and prostate, and it is detectable in high concentration in tears, saliva and breast milk [20]. The pharmacological half-life of minocycline is approximately 18 hours. Thus, high and frequent dosing is required.

Preliminary data on the efficacy of minocycline in patients with MS are promising. In an ongoing clinical trial of minocycline in a relatively small number of patients with relapsing-remitting MS, study participants received oral minocycline 100 mg twice daily for 6 months after a 3-month run-in period [21]. A 30-month treatment extension is ongoing. The preliminary results of this trial demonstrated that minocycline therapy reduces gadolinium-enhancing lesions on magnetic resonance imaging (MRI) [21]. Fortunately, no serious adverse effects of minocycline in MS have been reported thus far.

As our study in EAE animals shows, pharmacotherapy with long-circulating PEG minocycline-liposomes would substantially lower the total amount of minocycline administered to patients, yet provide similar clinical effectiveness. Thus, the risk of potential side effects of minocycline could be minimized. In our experiments, PEG minocycline-liposomes provided a sustained clinical benefit when given every five days, but not when given less frequently. The total expression of MMP-9 within the CNS and by splenocytes, a robust biomarker of minocycline activity, was also equally effected by daily minocycline treatment and injections with PEG minocycline-liposomes every five days. Interestingly, the expression of MMP-9 by CNS-infiltrating leukocytes was similar in all treatment groups, indicating that there is an absolute requirement for a certain level of MMP-9 expression by leukocytes to migrate across the blood-brain barrier, and to gain access to areas of inflammation. The absolute number of infiltrating cells was decreased.

Our present data emphasize the potential role of PEG liposomes as a drug delivery system for pharmaceuticals for CNS autoimmune diseases. Our data also indicate that inhibition of MMP-9 remains a promising treatment target in patients with MS.

## Materials and Methods

### Preparation of long-circulating PEG–liposomes

A lipid solution was prepared in ethanol, containing 100 mM dipalmitoyl phosphatidylcholine (DPPC), 8.1 mM PEG 2000 distearyl phosphatidylethanolamine (DSPE) (Lipoid GmbH, Ludwigshafen, Germany) and 54 mM cholesterol (Sigma, Poole, UK) in a molar ratio of 1.85∶0.15∶1.0. The lipid solution was transferred to a round-bottom flask, and a lipid film was created by rotary evaporation. The film was hydrated with a solution of 120 mM calcium chloride and 120 mM sodium acetate (MP Biomedicals, Inc., Eschwege, Germany). The resulting lipid dispersion was sized by multiple extrusions through polycarbonate filter membranes. Unencapsulated calcium chloride and sodium acetate was removed by dialysis. Mean particle size was determined by dynamic light scattering with a Malvern 4700 system (Malvern, Worcestershire, UK). The diameter of the liposomes was determined to be 100–110 nm with a polydispersity index below 0.2. To 8 ml liposomal formulation, 80 mg minocycline hydrochloride (MP Biomedicals Inc.) was added. This mixture was incubated at 50°C for 15 minutes. Phospholipid content was determined by phosphate assay [22] in the organic phase after extraction of liposomal preparations with chloroform. The aqueous phase after extraction was used for reversed-phase HPLC-determination of the minocycline content.

### In vitro assessment of mononuclear cells

Peripheral blood was aseptically collected from healthy volunteers by standard venipuncture into vacuum tubes containing sodium heparin as anticoagulant. PBMCs were isolated by Ficoll-Hypaque (Pharmacia, Karlsruhe, Germany) density gradient centrifugation and resuspended (2×10^6^/ml) in complete culture media consisting of RPMI 1640 medium supplemented with 10% human serum, 2 mM glutamine (Sigma-Aldrich, Munich, Germany), 100 U/ml penicillin, and 100 IE/ml streptomycin (Invitrogen, Karlsruhe, Germany). PBMCs were cultured for 24 h at 37°C and 5% CO_2_ in the presence of IL-2 (10 ng/ml; Sigma-Aldrich). Afterwards, PEG-liposomes were added and supernatants were obtained 1 and 6 h later. Informed written consent was obtained from all healthy volunteers, and all study procedures were approved by the Institutional Review Board at the Heinrich Heine University.

### Gelatin zymography

Sodium dodecyl sulfate-polyacrylamide gel electrophoresis (SDS/PAGE) zymography was performed for determination of gelatinase activity as described before [5], [11]. Supernatants were mixed 1∶1 with Tris/glycine SDS sample buffer (Novex, CA), and samples were applied to a 10% (w/v) polyacrylamide resolving gel containing 0.1% SDS and 0.1% gelatin type A from porcine skin (Sigma-Aldrich). Stacking gels were 5% (w/v) polyacrylamide. After electrophoresis gels were washed in renaturing buffer (Novex) containing Triton X-100 to remove any SDS and incubated in developing buffer (Novex) for 18 hours at 37°C. Gels were stained for 6 hours in 30% methanol / 10% acetic acid containing 0.5% (w/v) Coomassie Brilliant Blue G-250 and destained in the same buffer without dye. Gelatinase activity was detected as unstained bands on a blue background representing areas of gelatin digestion. As a negative control, gels were incubated with 10 mol/L *N*-ethylenediaminetetraacetic acid (EDTA) prior to the activation of gelatinases in parallel. Images of gels were captured by scanning on a flatbed scanner. A standard curve was obtained for densitometric quantitation of MMP activity using purified MMP-2 and -9 (Chemicon, CA). In each sample, MMP-9 proteolytic activity was calculated using an electrophoretic gel lane calculation software after image inversion.

### Mice

Female C57BL/6 (B6) mice were purchased from the Jackson Laboratory (Bar Harbor, ME). The use of mice in these experiments was approved by the Institutional Animal Care and Research Advisory Committee at the University of Texas Southwestern Medical Center at Dallas.

### Experimental autoimmune encephalomyelitis

EAE was induced in groups of five 6–10 week old female C57BL/6 mice by immunization with 200 µg of myelin oligodencrocyte glycoprotein (MOG) petide (p) 35–55 (BioSource International, CA) in an emulsion with Complete Freund adjuvant (CFA) containing 4 mg ml^−1^ of heat-inactivated *Mycobacterium tuberculosis* H37Ra (Difco Laboratories, MI). On the day of immunization and 48 hours later, C57BL/6 mice were injected with 200 ng *pertussis* toxin (PTx, List Biological Laboratories, Inc., CA) in phosphate buffered saline (PBS) i.p. Mice were examined daily for clinical signs of EAE and scored as follows: 0: No paralysis; 1: Loss of tail tone; 2: Hindlimb weakness; 3: Hindlimb paralysis; 4: Hindlimb and forelimb paralysis; 5: Moribund or death [23]. All experiments were repeated twice.

### Pharmacotherapy

For EAE treatment, the mice were treated with i.v. injections of PEG minocycline-liposomes (50 µg/g body weight [BW] in 100 µl of PBS) every five days. Empty PEG-liposomes (50 µg/g BW in 100 µl of PBS), i.p injections of minocycline (50 µg/g BW in 100 µl of PBS) every five days, and not pharmacological treatment constituted negative controls. Daily i.p. injections of minocycline (50 µg/g BW in 100 µl of PBS) was the positive control. In one dosing experiment, a single dose of i.v. injection of PEG minocycline-liposomes or PBS (control) was given at disease onset.

### Histopathological evaluation

Inflammatory infiltrates in the brain were quantified by haematoxylin and eosin (H&E) staining, as well as immunohistochemistry using antibodies against CD3, CD11b, and MMP-9 on days 20 after active immunization according to published methods [11], [24]. Selected brain, thoracic and lumbar spinal cord sections were evaluated by an examiner blinded to the treatment status of the experimental animals. Three mice per treatment group were examined. The mice selected for histopathological evaluation had the mean disease score of their respective treatment group.

### Preparation of CNS mononuclear cells

Mononuclear cells were isolated from brains and spinal cords as described [25]. CNS tissue was passed through a cell strainer (70 µm). After centrifugation, cells were resuspended in 37% Percoll (GE Healthcare, WI), centrifuged at 2,118 *g* for 15 min, and washed. To prepare cells for experiments, pooled CNS samples were washed twice with 37% Percoll, resuspended in 30% Percoll, and layered over 70% Percoll. Cells harvested from the gradient interface were washed and resuspended in culture medium consisted of RPMI 1640 (Invitrogen, CA) supplemented with L-glutamine (2 mM) (Mediatech Inc. VA), sodium pyruvate (1 mM) (Mediatech), non-essential amino acids (0.1 mM) (Mediatech), penicillin (100 U ml^−1^) (Mediatech), streptomycin (0.1 mg ml^−1^) (Mediatech), 2-mercaptoethanol (5×10^−5^ M) (Mediatech) and 10% (v/v) fetal bovine serum (Mediatech) for further analysis.

### Enzyme immunoassay

Supernatants from splenocytes and CNS mononuclear cells cultured with MOG_p35–55_ (2 ug/ml) were used for analysis. The concentration of total MMP-9 (pro-MMP-9, active MMP-9, and tissue inhibitor of matrix metalloproteases [TIMP]-1-complexed MMP-9) in cell culture supernatants was determined by Quantikine Mouse MMP-9 (total) Immunoassay according to the manufacturer's instructions (R&D Systems, MN). The results of ELISA experiments are expressed as an average of triplicate wells ±standard deviation (SD). A SOFTmax ELISA plate reader and software was used for data analysis (Molecular Devices, CA).

### Statistical analysis

The means of two normally distributed samples were compared by Student t-test. All other statistical comparisons between groups were examined using one-way multiple range ANOVA test for multiple comparison. P-values<0.05 were considered significant. Data are shown with SD of the mean.
